# Necrobiosis lipoidica arising on an old burn scar in a patient with Hashimoto's thyroiditis^⋆^

**DOI:** 10.1016/j.abd.2020.10.022

**Published:** 2022-06-14

**Authors:** Shohei Igari, Mayu Sato, Toshiyuki Yamamoto

**Affiliations:** Department of Dermatology, Fukushima Medical University, Fukushima, Japan

Dear Editor,

A 58-year-old female visited our department, complaining of asymptomatic skin lesions on the lower legs, which had appeared two years previously. She did not have diabetes; however, she had been diagnosed as having a goiter at almost the same time as when the skin lesions began and were under follow-up. Physical examination showed several well-circumscribed waxy brownish infiltrated plaques with elevated borders on the bilateral shins (Fig. 1A and 1B). Initial lesion arose on a burn scar, which had originally been caused by a Japanese electric foot warmer, that is used in the bed in winter. Thereafter, similar lesions were increased in number in the surrounding areas and spread to another lower leg. Laboratory examination showed normal liver and renal function; however, anti-thyroglobulin antibody (209.2 IU/mL; normal <28) and anti-thyroid peroxidase antibody (269.1 IU/mL, normal <16) were elevated. Thyroid Stimulating Hormone (TSH), TSH receptor antibody, and free T3 and T4 thyroid hormones were all within normal limits. Histological examination revealed necrobiotic changes of collagen in the dermis, surrounded by granulomatous inflammatory reactions composed of lymphocytes, histiocytes, and multinucleated giant cells (Fig. 2A). Of note, lymphoid aggregates were observed at the periphery of the degenerated collagen in the lower dermis. Immunohistological examination showed intense expression of CD3+ T-cells (Fig. 2B) and CD20+ B-cells (Fig. 2C). High endothelial venules-associated pNAd (MECA-79) epitopes were observed within the lymphoid aggregates (Fig. 2D), and CXCL13 positive cells were scattered within the lymphoid clusters.

**Figure 1:**
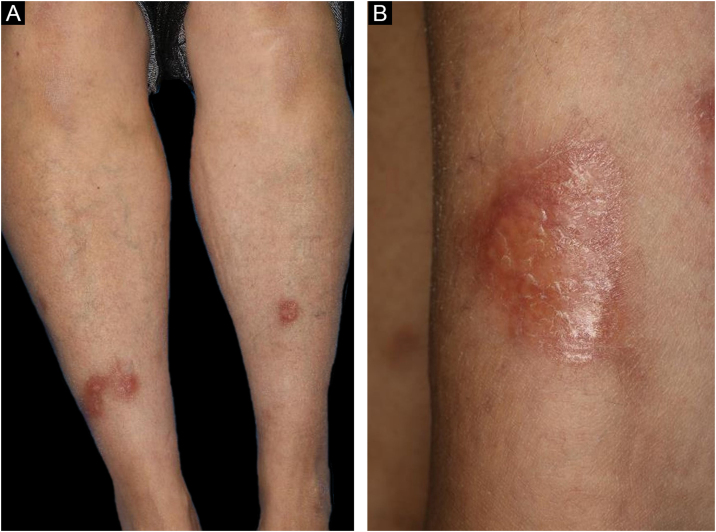
(A), Multiple, waxy brownish plaques on the bilateral shins with slightly elevated erythema at the periphery. (B), Close-up view.

**Figure 2:**
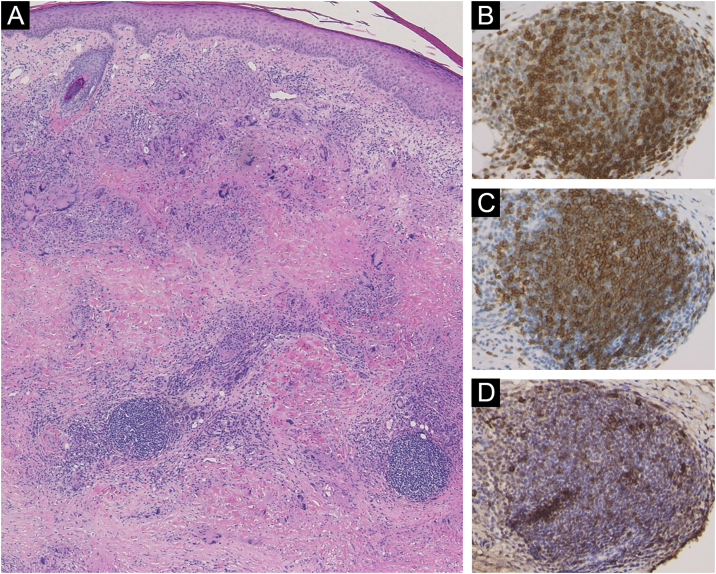
(A), Histological features showing horizontally arranged palisading histiocytes and multi-nucleated giant cells surrounding degenerated collagen in the dermis. Immunohistological examination showed that the lymphoid aggregates were positively stained for CD3 (B), CD20 (C), and pNAd (D).

The most common disease associated with Necrobiosis Lipoidica (NL) is diabetes mellitus, which was observed in 43% of the patients with NL, while thyroid disorders were detected in 15%.^1^ In the present case, NL and thyroid disease developed almost simultaneously. However, hormonal effects on the development of NL were unexpected because thyroid hormone levels were normal. Our patient developed NL as an initial lesion on the site of an old burn scar, which she received 40 years previously, then increased in number on the nearby areas unassociated with a burned scar. Burned sites undergo a reduction of immunity and thus become immunocompromised districts, where the immune behavior is compromised forever.^2^ Alternatively, NL occurred on the old scar fortuitously.

Another unique point in the present case is the histological feature of lymphoid follicles in the biopsied specimen. Lymphoid follicle-like structures were reported in the lesional skin of NL at a frequency of 11% (34 of 310 cases).^3^ Ectopic lymphoid neogenesis is associated with the development of high endothelial venules and is mediated by homing chemokines such as CXCL13. IL-17 and IL-23 are associated with the development of lymphoid follicles.^4^ IL-17 causes induction of the lymphoid chemokine, CXCL13. In the present case, CD20 was detected in the center of the lymphoid follicles, whereas CD3 was observed diffusely. pNAd-positive cells were scattered within the lymphoid follicles. Recent studies have shown that IL-17 was abundantly detected in NL, which may induce granuloma formation by suppressing regulatory T-cells.^5^ Further studies are needed to clarify the significance of ectopic lymphoid follicles in the pathogenesis of NL.

## Financial support

None declared.

## Authors' contributions

Shohei Igari: Designed the study; performed the study and contributed to analysis and interpretation of data; wrote the drafting the manuscript; approved the final version of the manuscript.

Mayu Sato: Performed the study and contributed to analysis and interpretation of data; approved the final version of the manuscript.

Toshiyuki Yamamoto: Designed the study; revised the manuscript for important intellectual content; approved the final version of the manuscript.

## Conflicts of interest

None declared.
